# On-panel characterization of early-age dynamic contact stress in fresh dry-mix shotcrete and its relation to rebound

**DOI:** 10.1617/s11527-026-03226-x

**Published:** 2026-08-12

**Authors:** Jongbeom Kim, Marc Jolin

**Affiliations:** Department of Civil and Water Engineering, Centre de recherche sur les infrastructures en béton (CRIB), Université Laval, Québec, G1V 0A6 Canada

**Keywords:** Shotcrete, Rebound, Dynamic contact stress, Fresh-state characterization, Receiving-surface response, Fluid zone

## Abstract

Dry-mix shotcrete rebound depends not only on spray kinematics but also on the early-age response of the receiving-surface during placement. This paper presents a full-scale on-panel experimental approach to characterize this response and relate it to rebound within one controlled dry-mix shotcrete material–equipment system. In this study, the term *fluid zone* denotes a hypothesized transient, localized, low-resistance near-surface region beneath the spray axis, identified from a wet/glossy appearance and lower on-panel *dynamic contact stress (*${p}_{d}$*)* measured by a dynamic bead-impact test. A robot-mounted, 3D-printed magnetic catapult and high-speed imaging were used to measure ${p}_{d}$ on the panel at 0.5 s after the nozzle passage and again at 3 and 6 min. Full-scale dry-mix spraying tests with controlled nozzle motion and real-time mass/flow measurements were conducted using a reference mixture and two air-entrained mixtures over a range of placement consistencies represented by ${\left(w/b\right)}_{\mathrm{c}\mathrm{a}\mathrm{l}\mathrm{c}}$. Under the tested setup, ${p}_{d}$ at 0.5 s was consistently lower than later values, providing indirect mechanical evidence consistent with a rapidly evolving low-resistance near-surface response. Lower early-age ${p}_{d}$ was associated with lower rebound. Air entrainment shifted both ${p}_{d}$ and rebound downward, while higher ${\left(w/b\right)}_{\mathrm{c}\mathrm{a}\mathrm{l}\mathrm{c}}$ toward the upper end of the accepted placement range were associated with lower rebound. The proposed on-panel test provides a comparative indicator of early-age receiving-surface response during dry-mix shotcrete placement. Although broader validation is required (equipment, mixture design, etc.), this measurement method appears to be a significant improvement of our understanding of rebound mechanisms.

## Introduction

Shotcrete consists of spraying concrete or mortar at high velocity onto a surface using pneumatic energy [1, 2]. This self-compacting placement technique is well suited to mining, tunnelling, slope stabilization, concrete repairs, and other applications, as it offers rapid material placement and excellent mechanical properties, all the while avoiding the need for costly or intricate formwork. Shotcrete is therefore a well-established and widely used concrete placement method in construction practice [1–3]. However, this process generates rebound, defined as material that ricochets off the receiving-surface during placement, which can have significant effects on *sustainability* [4] as well as on the *in-place* material properties. Indeed, coarse aggregates and fibres tend to rebound more, increasing the relative binder content of the in-place material [5, 6]. For these reasons, the mechanisms involved in the rebound phenomenon remain important, and the reduction and control of rebound are still major industry concerns.

Shotcrete placement is a complex process involving the kinetic energy of the incident particles and the receiving-surface’s behaviour and properties, which together influence particle retention and rebound [6, 7]. The impact energy is generated by compressed air, which accelerates the concrete or mortar particles through the nozzle. In practice, however, the material output, the airflow, and the type of nozzle used all have significant effects on the *spray of material* exiting the nozzle. Ginouse and Jolin [3, 8, 9] and Ginouse et al. [10] demonstrated that the spray of material exiting the nozzle can be described by the *velocities* of the particles, their *angles* relative to the nozzle axis, and the *spatial mass flow* within the spray. While the energy-based approach and these spray-scale descriptions of the spray help explain how mixture design parameters (aggregate gradation, binder content, etc.) and placement parameters (airflow, spraying distance, nozzle type, etc.) affect rebound, further progress requires a clearer understanding of the behaviour of the receiving-surface and its effects on rebound. A mechanical measurement performed directly on the panel is therefore needed to connect the local response of the fresh receiving-surface with material adhesion or rebound during placement. Recent studies have further shown that spraying and adhesion govern how material is distributed and retained at the receiving-surface, while pneumatic conveying remains a critical component of overall shotcrete process performance [11, 12].

In its simplest form, the placement and build-up of a shotcrete layer is still described by considering the initial stages of spraying as high-velocity particles hitting a rigid substrate and then ricocheting, leaving in place a thin layer of cement paste [13–15] until the first aggregates can be captured. As spraying continues, the cement paste layer thickens and progressively captures more particles, reducing the initially high rebound to an average value that stays constant throughout the thickness build-up. Although useful, this description provides little quantitative information on the actual mechanisms and properties involved in particle capture and rebound. Some researchers have used different tests to characterize the fresh shotcrete substrate (e.g.: flat-head needle penetrometer or shear-vane tests) and reported useful relationships with rebound and build-up thickness for specific mixture designs [6, 22], but direct measurement of substrate response *during* active placement remains very limited. Future improvements in reducing rebound, through mixture design [16], equipment selection [10], or especially placement techniques [17], are therefore likely to depend on better *description*, *understanding,* and *characterization* of fresh shotcrete surface behaviour during placement. Recent modelling and process-optimization studies also reflect increasing interest in quantitative descriptions of shotcrete spraying and placement processes [18, 19].

The purpose of this paper is therefore to characterize the early-age response of the freshly placed dry-mix shotcrete substrate *during* placement and to relate this response to rebound. The study introduces an on-panel dynamic contact stress test that can be performed within 0.5 s after nozzle passage and compares the resulting ${p}_{d}$ values with measurements taken at 3 and 6 min. The method combines a robot-mounted magnetic catapult, high-speed imaging, and real-time mass/flow measurements to compare the local dynamic indentation response with rebound under controlled spray kinematics. While the investigative approach applies to both wet-mix shotcrete and dry-mix shotcrete, the latter is the focus of this paper, given the relative importance of rebound with this method. Given that nozzle type, airflow, nozzle-to-surface distance, impact angle, spray motion, and particle impact configuration all influence spray kinematics and measured response, these factors were intentionally kept constant. The objective is not to establish universal ${p}_{d}$ thresholds for all dry-mix shotcrete systems, but to evaluate whether early-age ${p}_{d}$ can serve as a comparative indicator of receiving-surface response within one controlled material–equipment system.

## Problem description

A range of parameters affecting rebound in dry-mix shotcrete has been studied over the past few decades [3, 7–10, 13, 20–26]. In particular, work at the particle scale and spray characterization studies have clarified the influence of nozzle type, airflow, and the spraying process on shotcrete rebound [3, 7–10, 26]. Despite this progress, significant rebound reduction in dry-mix shotcrete remains a persistent challenge. A gap therefore remains in understanding and characterizing the behaviour of the *fresh shotcrete substrate* during full-scale spraying.

**Fig. 1:**
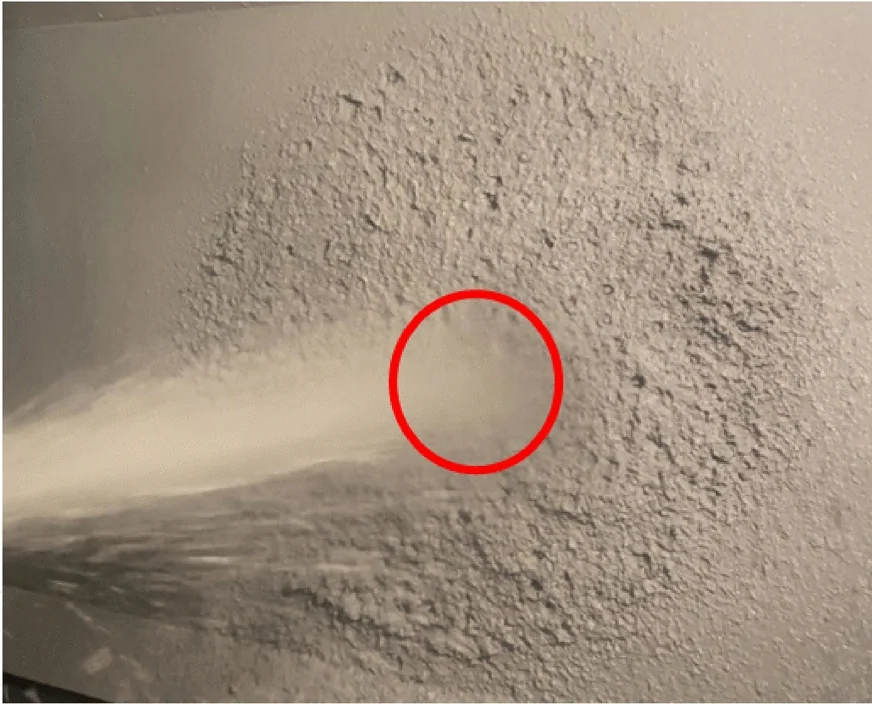
Close-up image showing the localized wet/glossy region beneath the spray axis referred to in this study as the fluid zone

Recent laboratory work at Université Laval suggests that the receiving-surface directly beneath the spray axis undergoes, for a brief moment, a localized change in response during active placement. Close-up images taken during spraying show a small wet/glossy region beneath the spray axis**.** There, coarse particles appear to penetrate more readily and remain embedded, whereas nearby areas appeared more granular and imprint-dominated (Fig. 1).

In the present study, the term *fluid zone* refers to a hypothesized transient, localized, low-resistance near-surface region directly beneath the spray axis during active placement. It is associated with two concurrent observations: (i) a localized wet/glossy surface appearance shown in Fig. 1, and (ii) lower bead-impact dynamic contact stress, or equivalently deeper bead penetration at Δt=0.5 s than at Δt=3 min and Δt=6 min under identical impact conditions. The present study does not provide a full-field geometric characterization of this region, but instead provides indirect mechanical evidence consistent with its transient presence under the tested full-scale spraying conditions.

Previous efforts to characterize this response during *full-scale spraying* were limited by the experimental difficulty of capturing substrate behaviour *during* active placement [21, 24]. Indeed, the effect of repeated particle impacts beneath the spray appears to temporarily reduce the apparent resistance of the near-surface layer of shotcrete [21, 23, 24]. Without being a strict rheological behaviour comparison, the shotcrete substrate response can be compared to that of fresh cast-in-place concrete in which a poker vibrator is inserted, leading to reduced apparent yield resistance [27, 28]. Noteworthy, in practice, highly skilled shotcreters have informally referred to this localized response as an *agitated puddle* [29] or *rebound-friendly zone*. This interpretation is used here only as a working hypothesis and does not imply that the region is a fully liquid phase or that its complete rheological state has been measured.

Capturing a mechanical signature of this short-lived response during full-scale spraying remains an important experimental challenge. The zone is hypothesized to arise from repeated high-energy particle impacts and to be influenced by factors such as material thixotropy and the spatial distribution of mass and energy in the spray. In the present study, the experimental scope was balanced against the control of the spraying process. Because equipment and placement variables strongly affect particle velocity, impact angle, and spatial mass distribution in the spray, the present program deliberately *limited the test parameters* and kept the main spray-kinematic factors constant. The resulting dataset is therefore intentionally limited but consistent, providing an initial basis for evaluating the test method and allowing direct comparison of receiving-surface response and rebound within one controlled full-scale configuration.

Dry-mix shotcrete also introduces unique challenges for characterizing its fresh behaviour. While classical concrete rheological models offer valuable insights into *material flow properties*, they do not directly capture the elastic deformation portion of the impact energy that is stored and restored as elastic strain energy, which is required to explain rebound [7]. Efforts have been made in the past to measure the rheology of shotcrete by preparing mortar mixtures that closely resemble dry-mix shotcrete in a mixer [25]. However, the properties of shotcrete undergo difficult-to-reproduce changes during the spraying and consolidation processes, which supports the use of full-scale spraying when the measured response depends on the placement process. Therefore, under placement-relevant conditions, alternative methods are needed to assess the receiving-surface response, and they must account for the *rapidly changing behaviour* of the dry-mix shotcrete substrate during placement. This consideration is crucial in the present study because fresh- material response may change rapidly (within seconds) during shotcrete placement.

The following sections present the substrate-characterization framework and the experimental work conducted at the *Shotcrete Laboratory* of Université Laval to test this concept. More broadly, the evaluation of fresh properties for dry-mix shotcrete should emphasize the importance of in situ measurements, preferably *during* and *after* spraying using full-scale equipment, so that the particularities of this pneumatic placement method are adequately captured [30].

## Fresh shotcrete substrate characterization

Armelin's description of rebound in dry-mix shotcrete involves three energy terms: the incident kinetic energy of the incoming particle (${W}_{1}$), the energy restituted by the substrate (${W}_{2}$), and the debonding energy (${W}_{d}$) [7]. Within this framework, rebound occurs when ${W}_{2}$ exceeds ${W}_{\mathrm{d}}$ [5, 7]. Each of these energy terms can be influenced by mixture composition, particle gradation, particle velocity, impact angle, and the spatial distribution of mass and energy in the spray. A subsequent model based on this framework showed that rebound can be rationally related to aggregate size, cement content, shooting consistency, and the presence of silica fume [31].

Since the penetration phase is linked to the kinetic energy of moving particles, it has been characterized using a *dynamic contact stress (*${p}_{d}$*)* parameter. Armelin [7] used ${p}_{d}$ within his rebound framework. The dynamic contact stress (${p}_{d}$) is defined from the penetration event as the ratio of the penetration work to the penetration volume. For a spherical projectile of mass m and impact velocity v that produces a penetration volume (${V}_{PEN}$),

1 $${p}_{d}=\frac{{W}_{PEN}}{{V}_{PEN}}=\frac{m{v}^{2}}{2{V}_{PEN}}$$

At fixed mass and impact velocity, lower ${p}_{\mathrm{d}}$ corresponds to a larger penetration volume and lower average resistance during penetration. The parameter characterizes the penetration phase. It does not independently determine the restituted energy (${W}_{2}$) or the debonding energy (${W}_{\mathrm{d}}$).

This formulation is based on plasticity and indentation mechanics [32–34]. Jolin [6] applied this approach experimentally to fresh shotcrete using a laboratory dynamic indentation method with spherical impactors. Armengaud et al. [20] later applied the same ${p}_{\mathrm{d}}$ definition using a 16 mm glass ball projected by a pneumatic launcher at different speeds. Ball speed was measured by radar, and ${p}_{\mathrm{d}}$ was evaluated 7 min after spraying.

These studies showed that delayed ${p}_{\mathrm{d}}$ measurements were sensitive to mixture design and could be related to rebound. However, they were not designed to measure the receiving-surface within the first instants after nozzle passage. The present method addresses this timing gap by performing the impact test directly on the panel 0.5 s after the nozzle passage and repeating the measurement at 3 and 6 min.

## Experimental program

### Laboratory setup for spraying shotcrete

The experimental program was designed to characterize the early-age receiving-surface response during placement and to relate that response to rebound. It also assessed whether the on-panel measurements were consistent with a transient, localized low-resistance zone beneath the spray axis. To support rebound-oriented comparison under controlled full-scale conditions, the base dry-mix shotcrete system, spraying equipment, robot-controlled nozzle-to-surface distance and impact angle, air supply, and impact-probe configuration were kept constant. This strategy reduced confounding variability in spray kinematics and impact conditions and supported comparisons of mixture designs, placement consistencies, and measurement time after nozzle passage in relation to early-age ${p}_{\mathrm{d}}$ and rebound. The following sections describe the experimental program and the methods used.

### Shotcrete equipment

An Aliva‑246 rotary‑barrel gun delivered the dry blend of materials through a 15 m long hose with a 38 mm internal diameter. The hose was attached to a hydromix nozzle fitted with a *Spirolet* tip; in this nozzle design, the water ring was located approximately 2 m upstream of the nozzle outlet, so that water and conveyed dry material had a longer mixing length before discharge, improving spray uniformity [1]. Water and liquid admixtures were supplied from a pressurized pneumatic tank to maintain stable water-line pressure.

**Fig. 2:**
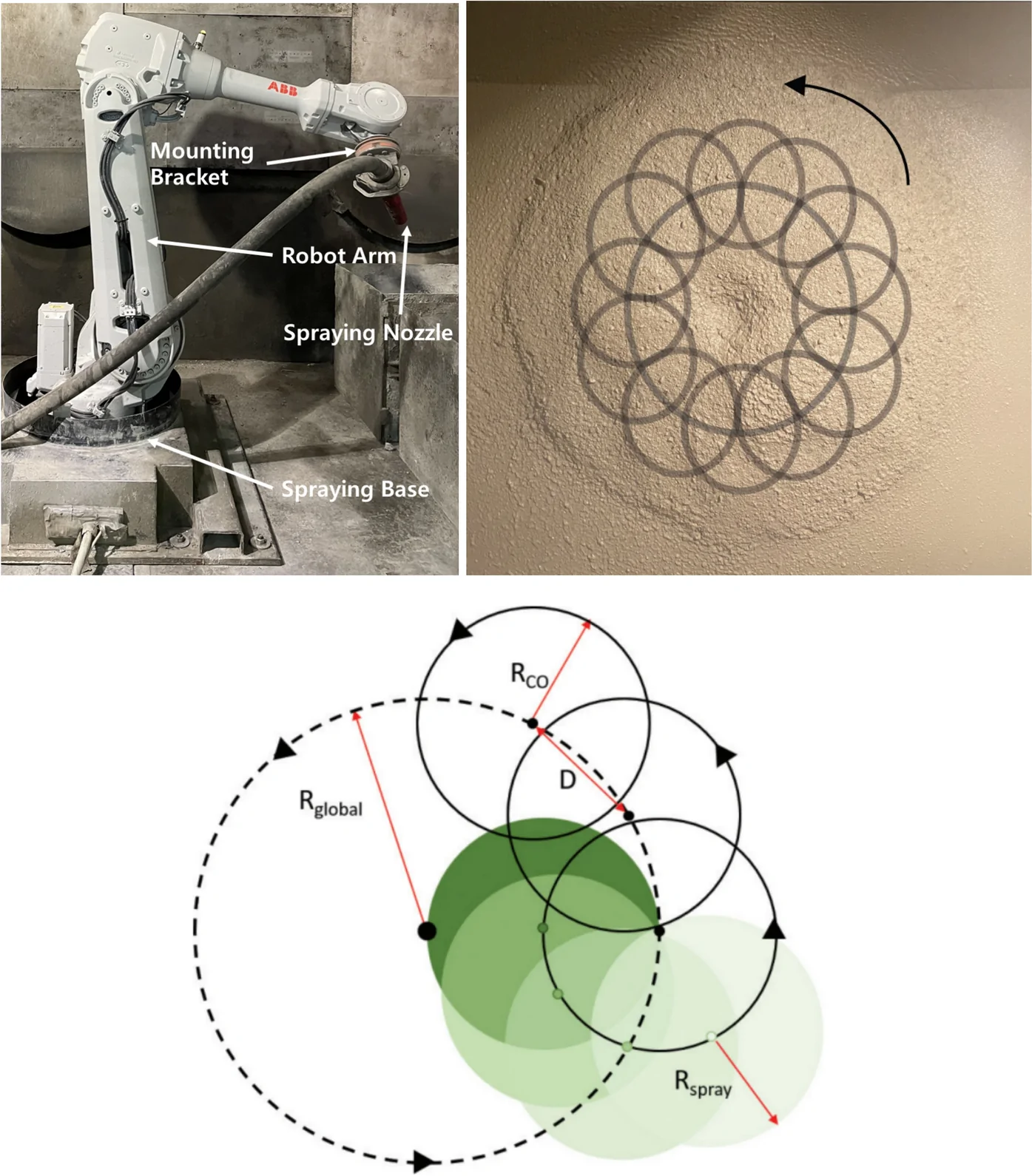
**a** ABB IRB-4600 industrial robot with shotcrete setup, **b** example of the nozzle trajectory (planetary motion) over the test panel, and **c** schematic definition of the planetary motion geometry adapted from Monfort et al. [17]

Spraying was performed against a 2 m × 1 m steel test panel using an ABB IRB-4600 six‑axis industrial robot (overall reach 2.05 m; payload 60 kg). The robot maintained a nozzle-to-surface distance of 1.0 m and kept the nozzle axis perpendicular to the panel. In this motion, the centers of the local circular paths progressed along a global circular trajectory, while the spray footprints overlapped progressively on the substrate (see setup and spraying pattern in Fig. 2). The rebound-oriented planetary motion adopted in the present program was characterized by a local-circle radius ${R}_{\mathrm{c}\mathrm{o}}$ of 110 mm and a spacing D of 110 mm between consecutive local-circle centers. The characteristic spray-footprint radius ${R}_{\mathrm{s}\mathrm{p}\mathrm{r}\mathrm{a}\mathrm{y}}$ was 110 mm, and the global radius ${R}_{\mathrm{g}\mathrm{l}\mathrm{o}\mathrm{b}\mathrm{a}\mathrm{l}}$ was 220 mm (Fig. 2c). By design, this motion avoided abrupt changes of direction and provided a controlled full-scale spraying pattern. Further details on the robot and the trajectory used can be found in Monfort et al. [17].

This spraying configuration was kept constant throughout the program because nozzle type, nozzle-to-surface distance, impact angle, airflow, and motion patterns govern particle velocity fields and spatial mass distribution in the spray.

**Fig. 3:**
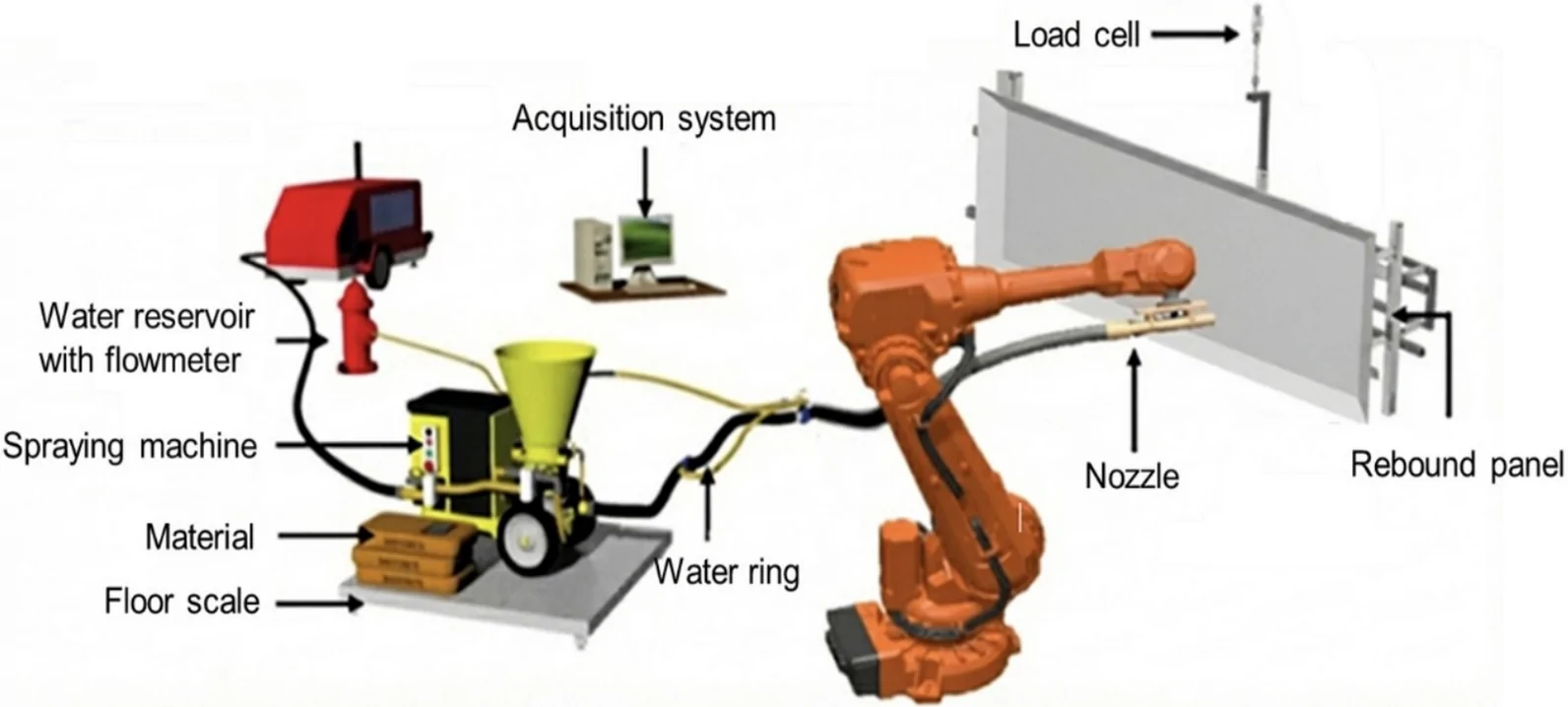
Schematic of the *Shotcrete Laboratory* setup and data-acquisition system used to record waterflow, air flow, dry-material feed, and deposited panel-mass

A computer recorded, in real time, the variables required to compute rebound during dry-mix spraying. The data-acquisition system logged (at 2 Hz) the water-flow, the compressed air flow, the cumulative mass of dry material fed from the machine (using a floor scale beneath the dry-mix machine), and the cumulative mass deposited on the test panel via a load cell. The water-line flow at the nozzle is monitored as a process quality-control measure (Fig. 3). Indeed, during spraying of the test panel, the shotcreter was instructed to keep the water setting unchanged, and the measured water-flow was checked over the rebound calculation interval to verify that no deliberate adjustment or instability occurred.

Rebound was evaluated over the full duration of each spraying session using mean mass rates obtained from the slopes of the cumulative-mass records over that interval as

2 $$\mathrm{R}\mathrm{e}\mathrm{b}\mathrm{o}\mathrm{u}\mathrm{n}\mathrm{d}\left(\%\right)=100\left(1-\frac{{\overline{\dot{m}} }_{dep}}{{\overline{\dot{m}} }_{dry}+{\overline{\dot{m}} }_{w}}\right)$$

where ${\overline{\dot{m}} }_{\mathrm{d}\mathrm{e}\mathrm{p}}$ [kg/s] is the mean mass rate of fresh material deposited on the test panel, obtained from the slope of the cumulative deposited-mass record over the spraying session; ${\overline{\dot{m}} }_{\mathrm{d}\mathrm{r}\mathrm{y}}$ [kg/s] is the mean dry-material feed rate, obtained from the slope of the cumulative dry-material feed record over the same interval; and ${\overline{\dot{m}} }_{w}$ [kg/s] is the mean water mass flow rate over that interval. The rebound calculation interval extended from the beginning of material deposition on the test panel to the end of spraying on the panel.

The mass flow rate of water was calculated from the measured volumetric water-flow as

3 $${\overline{\dot{m}} }_{w} = {\rho}_{w}{\overline{Q} }_{w}$$

where ${\overline{Q} }_{w}$ [L/s] is the mean volumetric water-flow and ${\rho}_{w}=1.00 kg/L$ is the water-equivalent density used to convert volumetric flow to mass flow.

Because all mean rates were evaluated over the same interval, this mass-rate formulation is equivalent to a cumulative-mass formulation based on total mass increments over the same interval.

### Measuring the ***dynamic contact stress (***${{\boldsymbol{p}}}_{\mathbf{d}}$***)***

In the present program, steel beads of 13.5 mm diameter and 10.0 g mass were launched onto the freshly sprayed substrate, and the dynamic contact stress (${p}_{\mathrm{d}}$) was evaluated from the measured impact velocity and the measured imprint geometry after bead extraction. For a bead of mass (m) impacting at velocity (v) and producing a penetration volume (${V}_{\mathrm{P}\mathrm{E}\mathrm{N}}),$ ${p}_{\mathrm{d}}$ was calculated using Eq. (1). Impact velocity was obtained from high-speed imaging.

Prior to the spraying tests, launcher calibration trials were performed to adjust the catapult release setting and to verify that the three beads were launched with comparable velocities and trajectories. High-speed imaging during ten calibration trials gave a mean bead velocity of 7.9±0.1 m/s, corresponding to a calculated incident energy of 0.31±0.01 J per bead. These calibration trials characterize the mechanical launch condition.

The nominal impact energy was compared with kinetic energies calculated from the coarse-particle velocities reported by Ginouse et al. [10] for a dry-mix Spirolet nozzle. In that study, particles larger than 5 mm near the spray axis reached axial velocities of approximately 28 to 30 m/s at 1.0 m from the nozzle outlet. The kinetic energy of an ideal spherical aggregate was estimated as ${W}_{a}={\rho}_{a}\pi {d}^{3}{v}^{2}/12$, where d is the aggregate diameter, v is the particle velocity, and ${\rho}_{a}$ is the aggregate density. Using ${\rho}_{a}=2650 \mathrm{k}\mathrm{g}/{\mathrm{m}}^{3}$, this velocity range gives calculated kinetic energies of approximately 0.23 to 0.26 J for a 7.5 mm spherical aggregate and 0.54 to 0.62 J for a 10 mm spherical aggregate. The 0.31 J nominal impact energy used in the present study falls between these two calculated kinetic energy ranges.

Because this impact energy was kept constant across all tests, variations in ${p}_{\mathrm{d}}$ primarily reflect changes in penetration volume and, therefore, in the resistance of the receiving-surface. For each extracted imprint, the measured imprint radius was denoted a, the measured imprint depth was denoted ${\delta}_{\mathrm{P}\mathrm{E}\mathrm{N}},$ and the bead radius was R=6.75 mm. The penetration volume ${V}_{\mathrm{P}\mathrm{E}\mathrm{N}}$ was calculated using a piecewise formulation.

For ${\delta}_{\mathrm{P}\mathrm{E}\mathrm{N}}<R,$ the penetration volume was calculated as the volume of a spherical cap. The spherical-cap height was calculated from the measured imprint radius as:

4 $$h=R-\sqrt{{R}^{2}-{a}^{2}}$$

and the corresponding penetration volume was

5 $${V}_{PEN}=\frac{\uppi {h}^{2}}{3}\left(3R-h\right)$$

For ${\delta}_{\mathrm{P}\mathrm{E}\mathrm{N}}\ge R$, the penetration volume was calculated as a spherical cap of height R, corresponding to a hemisphere, plus a cylindrical extension:

6 $${V}_{PEN}=\frac{2\uppi {R}^{3}}{3}+\left({\updelta}_{PEN}-R\right)\uppi {a}^{2}$$

This piecewise formulation was used because, under very fresh on-panel conditions, some measured imprint depths exceeded the bead radius and could not be described adequately by a spherical-cap approximation alone.

The 13.5 mm steel bead was selected as the smallest practical standardized spherical probe that, under the available catapult energy, still produced clearly visible and measurable imprints on the very fresh receiving-surface. Smaller beads would have produced smaller footprints that were more difficult to identify and measure reliably after extraction. Its size is of the same order as the coarse fraction of the base mix, but it was not intended to reproduce a single shotcrete aggregate particle on a one-to-one basis. Rather, it served as a controlled indentation probe for comparative on-panel ${p}_{\mathrm{d}}$ measurements. Steel beads and natural aggregates also differ in density, particle shape, surface roughness, contact geometry, and restitution behaviour. This choice also maintains continuity with earlier shotcrete bead-indentation approaches used to infer dynamic contact responses [6, 20].

At each measurement time, three identical steel beads were launched simultaneously in a single catapult shot; the three resulting imprints were measured using a caliper after bead extraction. The three individual bead-impact measurements were not interpreted as independent measurements from separate spraying sessions. Their dispersion therefore characterizes local variability within one catapult shot. Tests were executed at three specific moments after nozzle passage to capture the short-lived evolution of the surface: 0.5 s, 3 min, and 6 min. Here, Δt is defined as the elapsed time between passage of the spray axis over the test location and bead launch at that location. Lower values of ${p}_{\mathrm{d}}$ indicate lower average resistance during indentation and are consistent with a surface that facilitates particle embedment under the bead-impact condition. This on-panel impact test provides a rapid mechanical indicator of the receiving-surface response shortly after nozzle passage.

Because fresh shotcrete is locally heterogeneous, the three beads may interact with different near-surface configurations, including paste-rich regions, aggregate-rich regions, or near-surface coarse aggregate. No bead value was rejected on the basis of its magnitude or suspected aggregate contact. When an imprint was visible and its geometry could be measured, the corresponding result was retained. The individual bead values, mean, sample standard deviation, and coefficient of variation were then reported to describe the local response. This procedure was adopted to limit subjective data removal and to represent the heterogeneity of the fresh shotcrete surface under a common launch condition.

When ${p}_{\mathrm{d}}$ at Δt=0.5 s is lower than the values measured at 3 and 6 min under identical impact conditions, and when this occurs in the localized wet/glossy region beneath the spray axis, it indicates that the receiving-surface offered lower dynamic indentation resistance shortly after nozzle passage. This behaviour is consistent with the transient fluid zone hypothesis as defined in this study.

#### 3D-printed catapult

**Fig. 4:**
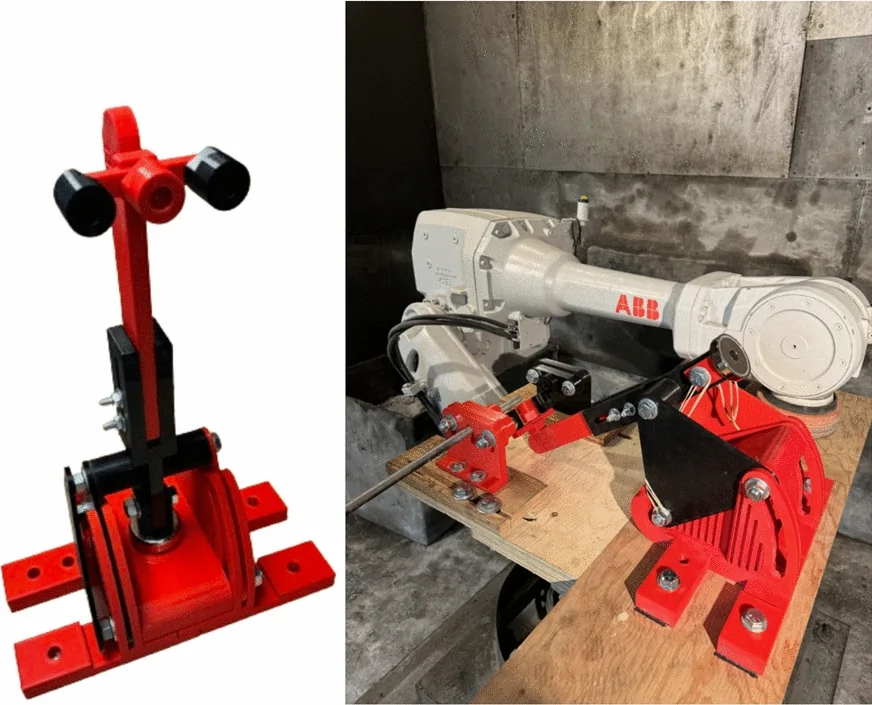
**a** 3D-printed magnetic catapult and **b** catapult mounted onto the robot’s wrist

To capture the short-lived early-age response of the receiving-surface beneath the spray axis, a 3D-printed catapult (Fig. 4a) was created and used in the laboratory. It uses a magnetic latch to release the three steel beads toward the freshly sprayed panel. The bead velocity and launcher calibration procedure are described in Sect. 4.3.

The catapult-to-panel distance, measured from the bead exit point to the panel surface, was 1.2 m and was kept constant throughout the program. Because of the mounting arrangement on the robot wrist, this distance differed from the 1.0 m nozzle-to-surface distance used for spraying. Within this mechanical constraint, the catapult was aligned as closely as possible with the panel normal, and high-speed video was used to verify that the bead trajectories were approximately perpendicular to the panel surface. This fixed three-bead launch configuration provided comparable impact orientation for all tests (Fig. 4a).

To minimize the delay between nozzle passage and bead launch, the catapult was mounted on a small platform attached to the robot’s wrist next to the nozzle. This arrangement allowed precise positioning of the catapult and triggering of the shot at Δt=0.5 s after passage of the spray axis over the test location (Fig. 4b). This setup reduced operator influence and allowed ${p}_{\mathrm{d}}$ to be measured at Δt=0.5 s.

After each catapult shot, retained beads were recovered from the imprints. When a bead was no longer retained at the impact location, the remaining visible imprint was located and measured. The catapult was then reloaded, and the robot’s wrist was moved slightly to adjacent locations on the same sprayed region so that additional tests could be conducted without overlapping the previous imprints. Tests at Δt=3 min and Δt=6 min were therefore obtained for comparison.

**Fig. 5:**
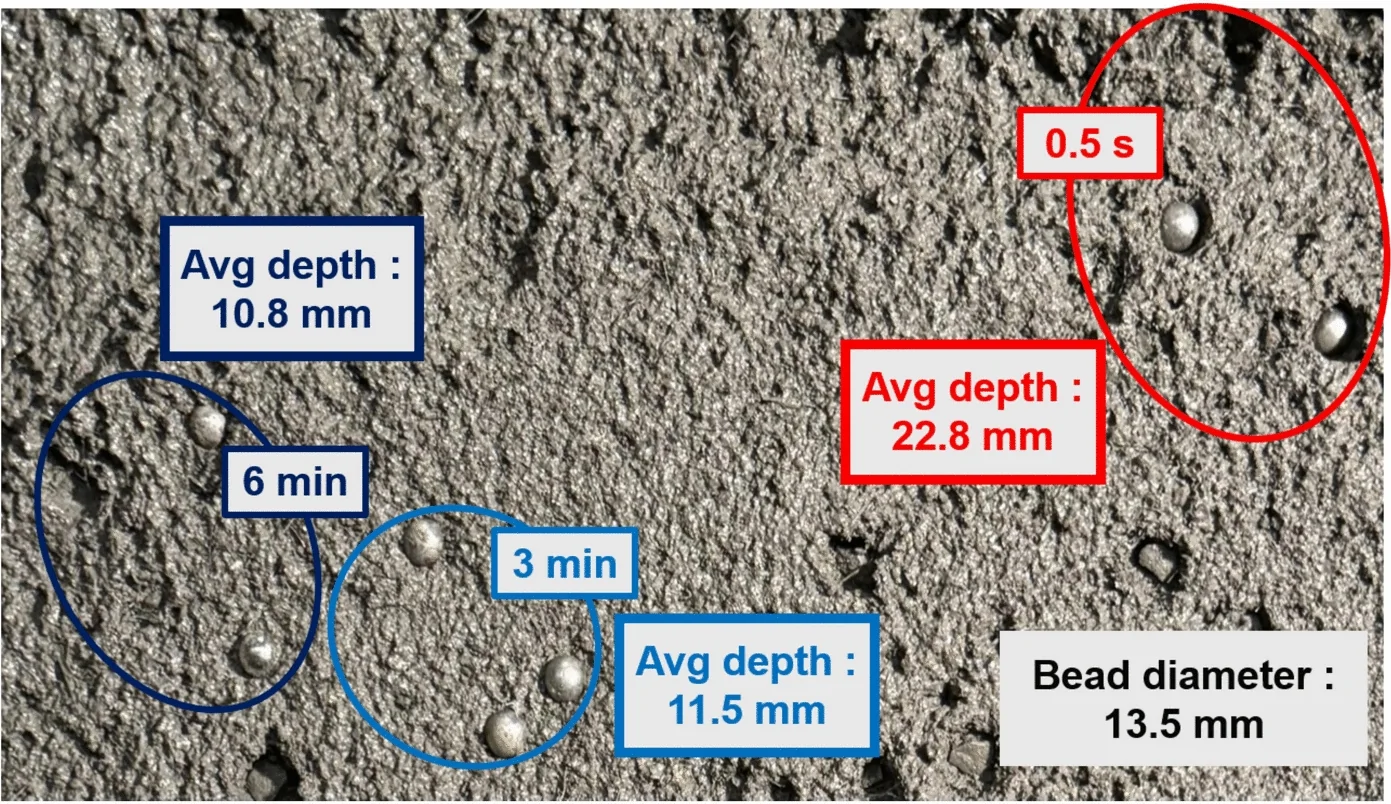
Bead-impact regions for the AEA-Ex-W condition at Δt=0.5 s, 3 min, and 6 min after nozzle passage. The circles identify retained beads and visible imprints left after bead extraction or when the bead was no longer retained at the impact location

Fig. 5 shows a visual example of the bead-impact regions for the AEA-Ex-W condition. The circles identify the bead-impact regions at each measurement time and include both beads retained at the impact location and visible imprints left after bead extraction or when the bead was no longer retained at the impact location. The reported depths are mean imprint depths from the three local measurements at each time and were obtained using a caliper. At 0.5 s, the measured imprints showed an average depth of 22.8 mm, roughly twice the depth measured a few minutes later. At 3 min, the mean imprint depth was 11.5 mm, and at 6 min, it was 10.8 mm. The larger imprint depths shown here are therefore consistent with lower resistance at 0.5 s. Because several measured imprint depths exceeded the bead radius, the corresponding ${V}_{\mathrm{P}\mathrm{E}\mathrm{N}}$ values were calculated using the formulation described in Sect. 4.3. For the 0.5 s AEA-Ex-W example, the imprint depth corresponded to ${\delta}_{PEN}/R\approx 3.4$, where R is the bead radius. This illustrates that a simple spherical-cap approximation was insufficient for some very fresh conditions and supports the use of the piecewise ${V}_{\mathrm{P}\mathrm{E}\mathrm{N}}$ formulation. The sharp evolution from 0.5 s to 3 min is consistent with a rapid increase in near-surface resistance after the spray moved away from the test location. The smaller gap between 3 and 6 min suggests that most of the changes occurred within the first few minutes.

The mixtures and placement consistencies used in the test program are described in the next section.

### Materials and mixture design

All mixtures, including the one shown in Fig. 5, were based on the same proprietary commercial prepackaged dry-mix shotcrete mixture complying with ACI PRC‑506‑22 Gradation No. 2 for dry materials [1], consistent with the dry-mix shotcrete particle-size distribution recommendations experimentally examined by Jolin and Beaupré [35]. The base aggregate–cementitious system was kept nominally constant, while the mixture design and placement consistency were varied as described below. The 30 kg-bag mixture consists of oven‑dried, graded aggregates (0–10 mm) and cementitious constituents (Table 1). Immediately before spraying, bags were emptied into the hopper of an Aliva‑246 machine. Potable municipal water was used as mixing water and supplied from a pressurized tank to the water line of the equipment.

**Table 1:** Typical composition of the base prepackaged dry-mix shotcrete used in this study

Mix*	OPC cement		Sand		Coarse aggregate (10 mm)		Silica fume (%, by mass of cement)		Placement consistency
	wt.%	kg/m^3^	wt.%	kg/m^3^	wt.%	kg/m^3^	wt.%	kg/m^3^	
M1–M3	24	493	63	1288	13	266	8	39	*Wet-Normal*

*The [kg/m^3^] values are calculated assuming a w/b=0.40 and an air content of 5%. They describe a nominal base material composition and should not be interpreted as measured in-place shotcrete proportions for each spraying session

The use of a single prepackaged dry-mix was deliberate. At this stage, the objective was to determine whether early-age on-panel ${p}_{\mathrm{d}}$ could discriminate receiving-surface response within one realistic full-scale material system while minimizing additional variability from changes in binder chemistry, aggregate grading, and base mix composition. The present data therefore support within-system comparisons rather than a broad survey across dry-mix shotcrete materials.

The intentional variables were limited to the mixture design and placement consistency, with the latter quantified using the calculated spraying-session water-to-binder ratio, ${\left(w/b\right)}_{\mathrm{c}\mathrm{a}\mathrm{l}\mathrm{c}}$.

The mixture identification used in this study was as follows:

(i) CTRL—reference condition with no intentional AEA;
(ii) AEA‑Std—supplier-standard air-entrained prepackaged dry-mix, corresponding to the manufacturer-recommended powder pre-blended AEA condition (for details, see Vézina [36]);
(iii) AEA‑Ex—the same mix as AEA-Std, with an additional Vinsol resin-based AEA introduced through the water line. This additional AEA solution was fed at 40 mL/L of spraying water at the nozzle, following the general approach reported by Beaupré et al. [37].

For each spraying session, ${\left(w/b\right)}_{\mathrm{c}\mathrm{a}\mathrm{l}\mathrm{c}}$ was calculated from the measured water-flow and dry-material feed rate, together with the nominal binder fraction of the prepackaged material. In mass-rate form, this calculation can be expressed as

7 $$(w/b)\mathrm{c}\mathrm{a}\mathrm{l}\mathrm{c}= \frac{{\overline{\dot{m}} }_{w}}{{\overline{\dot{m}} }_{\mathrm{d}\mathrm{r}\mathrm{y}}{f}_{\mathrm{b}}}$$

where ${\overline{\dot{m}} }_{w}$ is the mean water mass flow rate, ${\overline{\dot{m}} }_{\mathrm{d}\mathrm{r}\mathrm{y}}$ is the mean dry-material feed rate, and ${f}_{\mathrm{b}}$ is the nominal binder mass fraction of the dry material, including cement and silica fume. The ${\left(w/b\right)}_{\mathrm{c}\mathrm{a}\mathrm{l}\mathrm{c}}$ values were not independently verified by measuring the water content of freshly sprayed shotcrete. They should therefore be interpreted as calculated spraying-session descriptors, not as direct measurements of the local in-place water-to-binder ratio.

Given that in dry-mix shotcrete the shotcreter adjusts the amount of water added at the nozzle, it is common in laboratory testing to spray the same material more than once to generate different fresh in-place shotcrete *consistencies*. As is often the case in the laboratory, the material is first sprayed at a *normal consistency*, representative of a *mining-type application* where build-up and stability are prioritized. It is also sprayed at a *wet consistency*, representative of a *repair application* where reinforcing bars are present and finishing is required.

This wetter consistency creates more significant aggregate imprints and displays a more pliable material, often referred to as the *wettest stable consistency* [14, 20, 22, 30, 38]. This approach is also consistent with previous descriptions of wet shotcrete consistency aiming for the highest water content that allows for a stable application on the wall; the wet or glossy appearance of the surface is used as a practical visual cue [20]. This practice is consistent with a decades-old recommendation by Studebaker [14] suggesting that dry-mix shotcrete should be placed at its “wettest stable consistency”, as later laboratory and jobsite observations showed that it promoted lower rebound, proper in-place composition, and optimal placement quality [22, 30, 38].

In this study, the wet and normal consistencies labels describe practical settings well understood and easily reproduced by an experienced shotcreter in the industry and are not standardized consistency classes. All three mixtures above were sprayed under these different placement consistencies.

In the present study, no standardized sag, build-up, or sloughing test was performed. Instead, the stability of each spraying session used for analysis was checked for consistent deposition without visible sloughing or process interruption. Each spraying session used for analysis produced approximately 50 kg of fresh material on the test panel. The corresponding water-flow and panel-mass records described in Sect. 4.2 were used to confirm that the water setting was unchanged and that material continued to accumulate on the panel.

Taken together, the present program was structured around a limited number of intentionally varied factors within an otherwise controlled full-scale spraying configuration. Table 2 summarizes the factors that were kept constant and those that were intentionally varied.

**Table 2:** Experimental design: controlled factors and intentionally varied factors

Category	Factor	Levels/conditions in this study	Role in the design
Controlled	Base material	One commercial prepackaged dry-mix shotcrete mixture	Maintain a common material system
Controlled	Spraying equipment	Aliva-246 rotary-barrel gun, hydromix nozzle, and Spirolet tip	Maintain a common spray configuration
Controlled	Spray kinematics	Robot-controlled nozzle-to-surface distance, perpendicular nozzle axis, and programmed planetary motion	Reduce variability in spray kinematics
Controlled	Air supply	Fixed during the program	Reduce variability in particle acceleration
Controlled	Impact probe	13.5 mm steel beads, fixed launch velocity, and approximately perpendicular impacts	Standardize the ${p}_{\mathrm{d}}$ measurements
Varied	Mixture design	CTRL, AEA-Std, AEA-Ex	Assess effect of air entrainment
Varied	Placement consistency and ${\left(w/b\right)}_{\mathrm{c}\mathrm{a}\mathrm{l}\mathrm{c}}$	Normal and wet placement consistencies; session-specific ${\left(w/b\right)}_{\mathrm{c}\mathrm{a}\mathrm{l}\mathrm{c}}$	Assess effect of water addition
Varied	Measurement time	0.5 s, 3 min, 6 min	Examine temporal evolution after nozzle passage

### Data treatment and statistical reporting

**Fig. 6:**
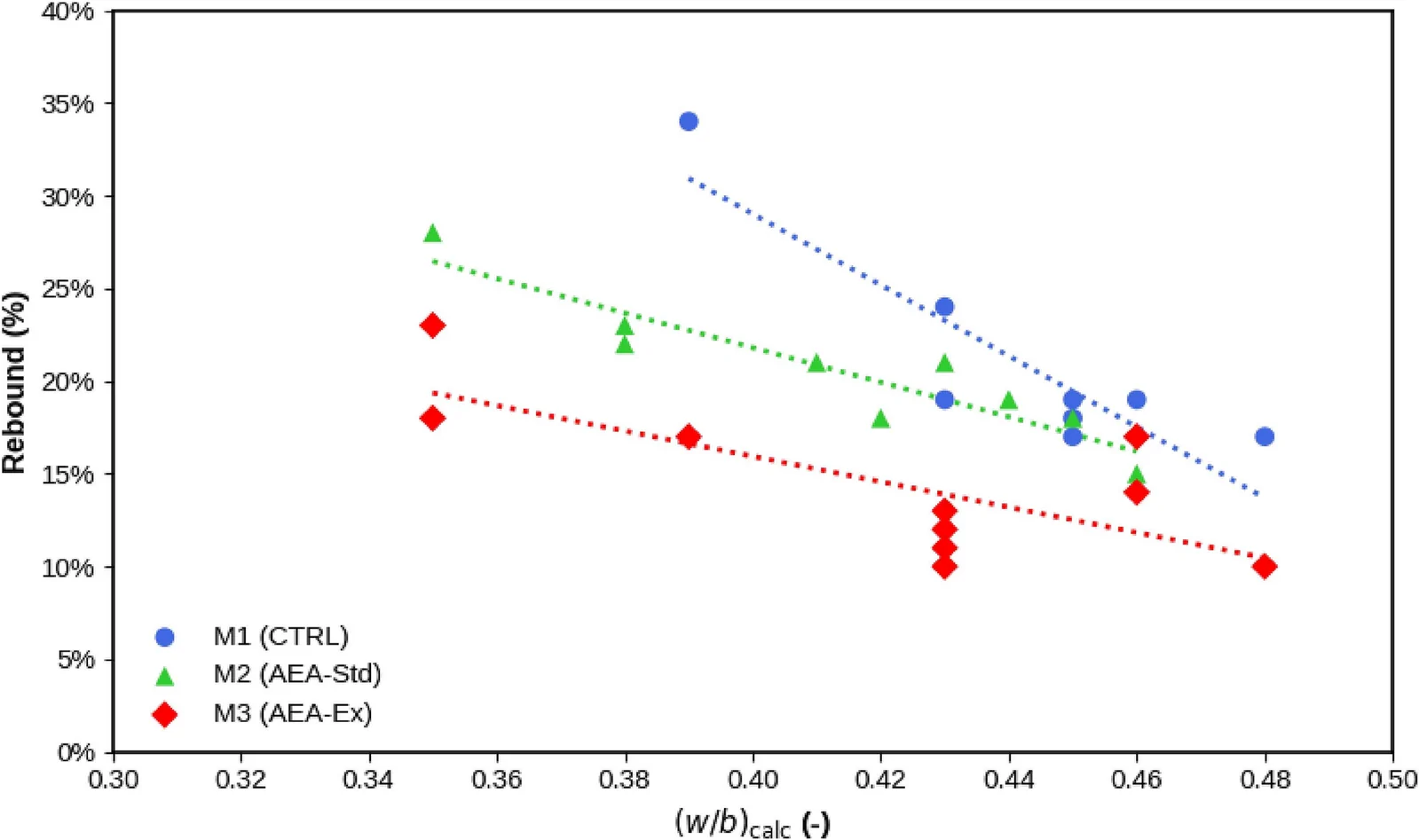
Rebound versus calculated spraying-session water-to-binder ratio, ${\left(w/b\right)}_{\mathrm{c}\mathrm{a}\mathrm{l}\mathrm{c}}$, for M1 (CTRL), M2 (AEA-Std), and M3 (AEA-Ex). Each point represents one spraying session. Dotted lines show separate descriptive linear fits for the three mixes

Accepted spraying sessions were treated as the independent observational units for the rebound–${\left(\mathrm{w}/\mathrm{b}\right)}_{\mathrm{c}\mathrm{a}\mathrm{l}\mathrm{c}}$ analysis shown in Fig. 6. Descriptive linear fits were calculated separately for CTRL, AEA-Std, and AEA-Ex, and the corresponding fitted slopes, coefficients of determination (${R}^{2}$), and 95% confidence intervals are reported in Table 3. A single-session removal sensitivity check was also performed by refitting each mixture-specific linear fit after excluding one session at a time. Given the limited number of sessions per mixture design, these fitted relations are interpreted primarily in a descriptive and comparative sense.

**Fig. 7:**
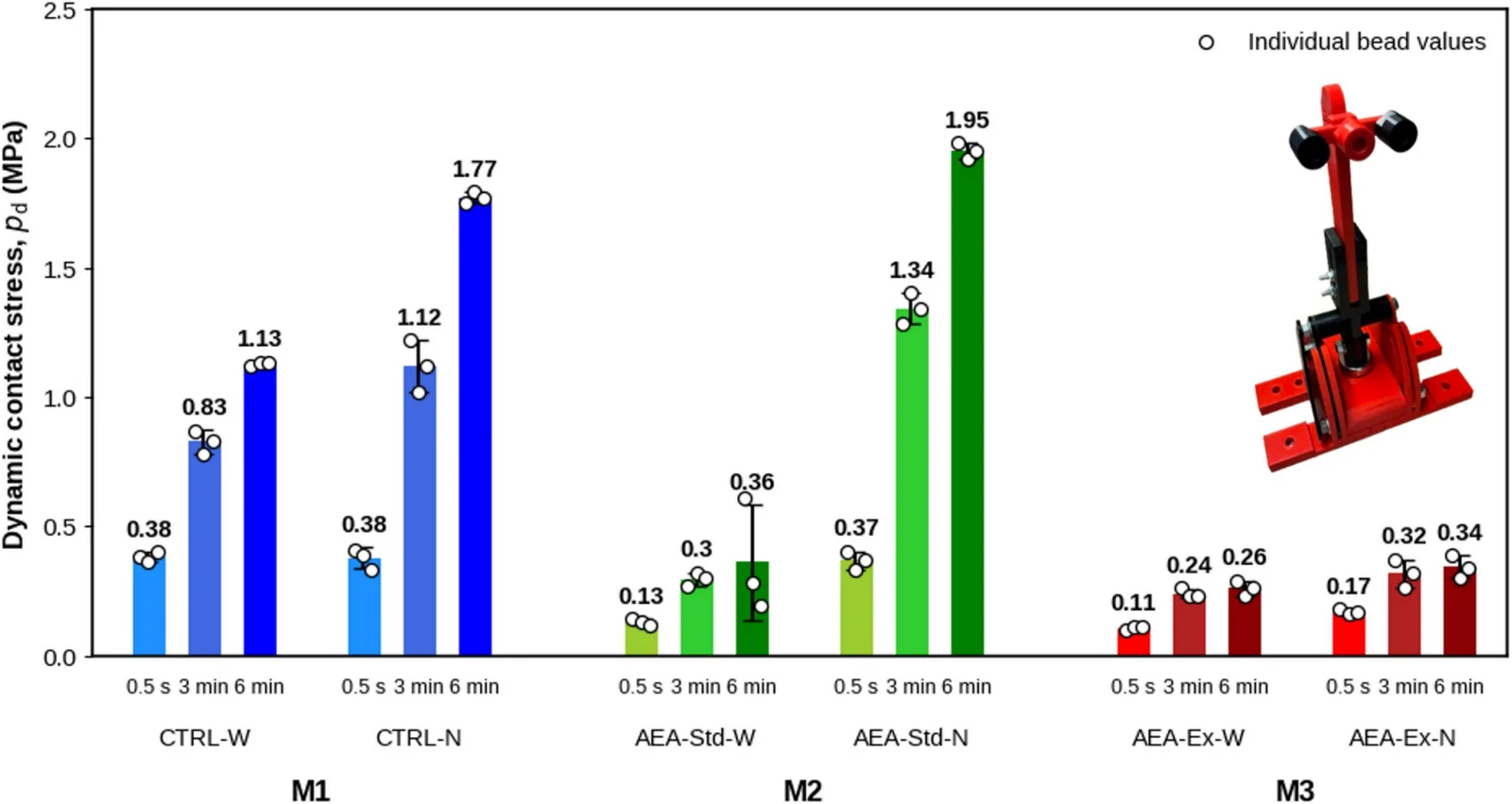
Dynamic contact stress (${p}_{\mathrm{d}}$) from on-panel bead impacts at 0.5 s, 3 min, and 6 min for CTRL, AEA‑Std, and AEA‑Ex under wet and normal consistencies. Individual points show the three bead-impact measurements from one catapult shot, and bars show the mean ± sample standard deviation

For Fig. 7, each bar represents the mean ± SD of three individual bead-impact measurements from a single catapult shot. These measurements describe local variability within one catapult shot and were not treated as measurements from separate spraying sessions. Therefore, no formal statistical significance test was applied to the Fig. 7 comparisons. Accordingly, the p_d_ values reported in Fig. 7 are interpreted as comparative indicators within the tested system rather than statistically generalizable material constants.

## Results

### Rebound in relation to placement consistency, ${\left({\boldsymbol{w}}/{\boldsymbol{b}}\right)}_{\mathbf{c}\mathbf{a}\mathbf{l}\mathbf{c}}$, and AEA

Figure. 6 plots rebound versus ${\left(w/b\right)}_{\mathrm{c}\mathrm{a}\mathrm{l}\mathrm{c}}$, where each data point represents an individual spraying session. Across the three mixtures, the same overall trend is observed: sessions with larger ${\left(w/b\right)}_{\mathrm{c}\mathrm{a}\mathrm{l}\mathrm{c}}$, corresponding to more water added at the nozzle, were associated with lower rebound.

The dataset included 27 sessions (CTRL: n = 8; AEA-Std: n = 9; AEA-Ex: n = 10). Separate descriptive linear fits showed negative rebound versus ${\left(w/b\right)}_{\mathrm{c}\mathrm{a}\mathrm{l}\mathrm{c}}$ slopes for all three mixes (Table 3). In all three cases, the 95% confidence intervals for the fitted slopes excluded zero, and a single-session removal sensitivity check showed that the fitted slope remained negative when any single session was excluded.

Relative to CTRL, the supplier-standard AEA condition (AEA-Std) showed lower rebound at comparable ${\left(w/b\right)}_{\mathrm{c}\mathrm{a}\mathrm{l}\mathrm{c}}$, and the enhanced AEA condition used in AEA-Ex showed an additional reduction. The clearest separation among the three mixes occurs in the mid-range ${\left(w/b\right)}_{\mathrm{c}\mathrm{a}\mathrm{l}\mathrm{c}}$, approximately 0.35 to 0.45, which lies within the tested placement range obtained under the present setup (Sect. 4.4).

The negative slopes are consistent with the *wettest stable consistency* concept defined in Sect. 4.4, provided that the fresh layer remains stable.

In the present tests, AEA-Std and AEA-Ex occupied lower rebound ranges than CTRL at comparable ${\left(w/b\right)}_{\mathrm{c}\mathrm{a}\mathrm{l}\mathrm{c}}$, with AEA-Ex showing the lowest rebound values in the tested dataset. Air entrainment shifted the rebound–${\left(w/b\right)}_{\mathrm{c}\mathrm{a}\mathrm{l}\mathrm{c}}$ relation downward. The corresponding changes in early-age dynamic contact stress are examined in the next section.

**Table 3:** Descriptive rebound versus ${\left({\boldsymbol{w}}/{\boldsymbol{b}}\right)}_{\mathbf{c}\mathbf{a}\mathbf{l}\mathbf{c}}$ linear fits and single-session removal check for spraying sessions

Mix	n	${\left(w/b\right)}_{\mathrm{c}\mathrm{a}\mathrm{l}\mathrm{c}}$	Rebound (%)	Slope	Intercept	${R}^{2}$	95% CI for slope	Slope range after single-session removal
CTRL	8	0.39 to 0.48	17 to 34	− 191.4	105.6	0.786	− 291.3 to − 91.5	− 233.8 to − 88.9
AEA-Std	9	0.35 to 0.46	15 to 28	− 93.2	59.1	0.851	− 128.0 to − 58.4	− 99.7 to − 75.7
AEA-Ex	10	0.35 to 0.48	10 to 23	− 68.5	43.3	0.529	− 121.1 to − 15.8	− 82.3 to − 45.1

The fitted model for each mixture design was Rebound (%) = intercept + slope × ${\left(\mathrm{w}/\mathrm{b}\right)}_{\mathrm{c}\mathrm{a}\mathrm{l}\mathrm{c}}$. The slope range after single-session removal was obtained by refitting each fit after excluding one session at a time. These fits are used to summarize the observed trends within the tested range

### Dynamic contact stress (${{\boldsymbol{p}}}_{\mathbf{d}}$)

Using the procedure and the catapult described in Sects. 4.3 and 4.3.1, Fig. 7 presents the results for CTRL, AEA-Std, and AEA-Ex under wet (W) and normal (N) consistencies. Across the six mixture–consistency combinations, ${p}_{\mathrm{d}}$ rose sharply between 0.5 s and 3 min, then increased more slowly to 6 min. This highlights the importance of measuring ${p}_{\mathrm{d}}$ shortly after nozzle passage.

At 0.5 s, the mean ${p}_{\mathrm{d}}$ values varied with mixture and consistency. AEA‑Ex gave the lowest ${p}_{\mathrm{d}}$ values in both W and N settings. AEA‑Std-W showed a lower ${p}_{\mathrm{d}}$ than CTRL-W, whereas AEA-Std-N was close to CTRL-N at 0.5 s. Within each mix, the W series generally lay below its N counterpart at all times. These time histories show a quick rise from the state measured at 0.5 s after nozzle passage, then a slower increase to 6 min. At fixed bead mass and velocity, the lower ${p}_{\mathrm{d}}$ values at 0.5 s indicate larger penetration volumes and lower average dynamic indentation resistance than at 3 and 6 min.

These measurements show local bead-to-bead variability within each shot. At 0.5 s, the coefficient of variation ranged from 5.3 to 13.6% across the tested series. For every series, the highest ${p}_{\mathrm{d}}$ value measured at 0.5 s remained below the lowest value measured at 3 min. This separation indicates that the difference between the immediate and delayed responses was larger than the local bead-to-bead variability within each shot.

## Discussion

The present study evaluates receiving-surface response under full-scale dry-mix shotcrete placement rather than under simplified off-panel laboratory preparation. The combination of robot-controlled nozzle motion, on-panel measurement at Δt=0.5 s after nozzle passage, a robot-mounted catapult, and simultaneous mass/flow acquisition makes it possible to relate a short-lived mechanical response to rebound within the tested material–equipment system.

From a fresh-shotcrete characterization perspective, the proposed on-panel ${p}_{\mathrm{d}}$ test provides a receiving-surface response indicator that can be applied during full-scale dry-mix shotcrete placement. Unlike conventional post-spray penetration tests or off-panel rheology measurements, the method characterizes the receiving-surface response shortly after nozzle passage, when particle embedment and rebound may be governed by a rapidly evolving near-surface condition. Here, ${p}_{d}$ is used to compare receiving-surface response across mixture designs, placement consistencies, and measurement times after nozzle passage.

### Early-age (0.5 s) dynamic contact stress and evidence of a transient low-resistance zone

The results provide indirect mechanical evidence consistent with a transient low-resistance near-surface response beneath the spray axis during active placement. Dynamic contact stress measured on-panel at 0.5 s was lowest for AEA‑Ex in both wet (W) and normal (N) settings. AEA-Std-W also showed a lower ${p}_{\mathrm{d}}$ than CTRL-W, whereas AEA-Std-N remained close to CTRL-N. This trend is consistent with the rebound results in Fig. 6 at comparable ${\left(w/b\right)}_{\mathrm{c}\mathrm{a}\mathrm{l}\mathrm{c}}$, where AEA-Ex occupied the lowest rebound range and CTRL the highest range. These patterns indicate that lower early‑age ${p}_{\mathrm{d}}$ corresponds to larger penetration volume and lower average dynamic indentation resistance at fixed bead mass and velocity. This response is consistent with greater particle embedment and lower rebound.

The time history in Fig. 7 shows that ${p}_{\mathrm{d}}$ rose quickly between 0.5 s and 3 min and then increased more slowly to 6 min. For each mixture–consistency combination, the highest ${p}_{\mathrm{d}}$ value measured at 0.5 s remained below the lowest value measured at 3 min. This rise is consistent with rapid dissipation of the short-lived low-resistance response after the spray moved away from the test location. Placed within Armelin’s rebound framework, this result is most directly related to the penetration phase of the impact event. Since ${p}_{d}={W}_{\mathrm{P}\mathrm{E}\mathrm{N}}/{V}_{\mathrm{P}\mathrm{E}\mathrm{N}}$, lower ${p}_{d}$ under fixed bead mass and velocity reflects a larger penetration volume and lower average resistance during indentation. The test does not determine the complete rebound-energy balance, but the association between lower early-age ${p}_{d}$ and lower rebound is consistent with the energy-balance interpretation of rebound [5–7].

This interpretation is consistent with known spray kinematics [3, 8–10]. Particle velocities and mass flow peak near the spray axis and decrease toward the edges, which concentrates impact energy at the centre and may contribute to the localized response referred to here as the fluid zone.

For the present material–equipment system, and under the robot-controlled spraying configuration selected on the basis of previous rebound-focused work, the lowest-rebound stable placements were associated with ${p}_{\mathrm{d}}$ at 0.5 s near 0.11 to 0.17 MPa. This interval is therefore best understood as an observed range for the present configuration. It helps rank stable low-rebound conditions and provides a reference range for comparing air entrainment and placement consistency within a spraying configuration.

### Scope of the experimental design

The experimental design was intentionally limited. All tests were performed using one commercial prepackaged dry-mix shotcrete, one hydromix nozzle fitted with a Spirolet tip, one robot-controlled spraying configuration with constant nozzle-to-surface distance and perpendicular nozzle axis, one air-supply setting, and one bead probe size under approximately perpendicular impact. This design reduced confounding variability in spray kinematics and enabled direct comparison of air entrainment, placement consistency, and time after nozzle passage within one controlled full-scale setup. The ${p}_{d}$ range identified in this study should therefore be read as an operating range for this material–equipment system, not as a transferable threshold. Changes in material composition, aggregate grading, nozzle design, airflow, nozzle-to-surface distance, impact angle, particle-size distribution, or spray motion may change both the early-age receiving-surface response and the associated rebound.

### Recommendations for future work

Building on the controlled benchmark established here, future work should extend the on-panel ${p}_{\mathrm{d}}$ measurement method across additional dry-mix materials, aggregate gradings, nozzle types, air-supply levels, nozzle-to-surface distances, impact angles, spray trajectories, admixture strategies, and impact-probe conditions.

Additional independent spraying sessions should be used to quantify repeatability of the 0.5 s ${p}_{\mathrm{d}}$ measurement. Spatial mapping of early-age ${p}_{\mathrm{d}}$ would also help clarify the spatial extent and duration of the transient low-resistance response beneath the spray axis.

Direct measurements of aggregate velocities during spraying and high-speed imaging observation of the impact would improve the comparison between bead impact energy and the particle-impact energy distribution of the spray. In addition, combining on-panel ${p}_{\mathrm{d}}$ measurements with fresh rheology, air-void system, and microstructure measurements would help connect the penetration-related receiving-surface response with the broader rebound-energy framework. Together, these extensions would help assess the transferability of the observed ${p}_{d}$ range for rebound-oriented mixture and process evaluation.

## Conclusions

This study examined the early-age receiving-surface response of fresh dry-mix shotcrete within one controlled full-scale material–equipment system and related that response to rebound using an on-panel dynamic contact stress test. Within the scope of the present study, the following conclusions can be drawn.

1. Early‑age dynamic contact stress (${p}_{\mathrm{d}}$) measured at Δt=0.5 s after nozzle passage was consistently lower than at 3 min and 6 min, providing evidence consistent with a transient and rapidly evolving low-resistance near-surface response shortly after nozzle passage.
2. At 0.5 s, AEA‑Ex gave the lowest ${p}_{\mathrm{d}}$ values in both wet and normal placement consistencies. Within each mixture, the wet placement consistency generally gave lower ${p}_{\mathrm{d}}$ than the normal placement consistency.
3. Lower early‑age ${p}_{\mathrm{d}}$ was associated with lower rebound at comparable calculated spraying-session water-to-binder ratios, ${\left(w/b\right)}_{\mathrm{c}\mathrm{a}\mathrm{l}\mathrm{c}}$. This association supports the relevance of the early on-panel indentation response to particle embedment and rebound within the tested system.
4. Within the tested material–equipment system, the lowest-rebound stable placements were associated with ${p}_{\mathrm{d}}$ values at Δt=0.5 s near 0.11 to 0.17 MPa. This interval should be interpreted as an observed range for the present configuration, not as a universal threshold.
5. Air entrainment shifted the rebound–${\left(w/b\right)}_{\mathrm{c}\mathrm{a}\mathrm{l}\mathrm{c}}$ relation downward within the tested range. Among the tested mixtures, AEA-Ex showed the lowest rebound range.

Taken together, these outcomes show that on-panel ${p}_{\mathrm{d}}$ can be used as a comparative indicator of early-age receiving-surface response during dry-mix shotcrete placement. The method provides a full-scale characterization approach for linking mixture design, placement consistency, short-term surface evolution, and rebound. Broader validation across materials, aggregate gradings, nozzles, impact-probe configurations, spray trajectories, and independent spraying-session repetitions would help assess transferability. The proposed approach offers a basis for more rigorous characterization of fresh shotcrete placement mechanisms and for future rebound-oriented mixture and process optimization.

## Data Availability

Data will be made available on reasonable request.
